# Efficient Super-Resolution Method for Targets Observed by Satellite SAR

**DOI:** 10.3390/s23135893

**Published:** 2023-06-25

**Authors:** Seung-Jae Lee, Sun-Gu Lee

**Affiliations:** Korea Aerospace Research Institute, 169-84, Gwahak-ro, Daejeon 34133, Republic of Korea

**Keywords:** KOMPSAT-5, SAR remote sensing, satellite SAR, super-resolution, target response

## Abstract

This study presents an efficient super-resolution (SR) method for targets observed by satellite synthetic aperture radar (SAR). First, a small target image is extracted from a large-scale SAR image and undergoes proper preprocessing. The preprocessing step is adaptively designed depending on the types of movements of targets. Next, the principal scattering centers of targets are extracted using the compressive sensing technique. Subsequently, an impulse response function (IRF) of the satellite SAR system (IRF-S) is generated using a SAR image of a corner reflector located at the calibration site. Then, the spatial resolution of the IRF-S is improved by the spectral estimation technique. Finally, according to the SAR signal model, the super-resolved IRF-S is combined with the extracted scattering centers to generate a super-resolved target image. In our experiments, the SR capabilities for various targets were investigated using quantitative and qualitative analysis. Compared with conventional SAR SR methods, the proposed scheme exhibits greater robustness towards improvement of the spatial resolution of the target image when the degrees of SR are high. Additionally, the proposed scheme has faster computation time (CT) than other SR algorithms, irrespective of the degree of SR. The novelties of this study can be summarized as follows: (1) the practical design of an efficient SAR SR scheme that has robustness at a high SR degree; (2) the application of proper preprocessing considering the types of movements of targets (i.e., stationary, moderate motion, and complex motion) in SAR SR processing; (3) the effective evaluation of SAR SR capability using various metrics such as peak signal-to-noise ratio (PSNR), structural similarity index (SSIM), focus quality parameters, and CT, as well as qualitative analysis.

## 1. Introduction

Satellite synthetic aperture radar (SAR) has been the main instrument used to monitor specific targets because it can offer two-dimensional (2D) target scattering information at all times with all-weather imaging capability [1,2,3,4,5,6,7,8]. The scattering information of targets should be clearly recognizable by humans or machines to achieve reliable target monitoring performance because it reflects the physical characteristics (e.g., structure and shape), category (e.g., type and model), and states (e.g., movement and change information) of targets.

In general, the spatial resolution of satellite SAR images plays a crucial role in representing the scattering information of targets in the 2D image domain. As the spatial resolution of satellite SAR images improves, the scattering information of the targets becomes clearer [9,10]. This is because better spatial resolution makes the impulse response functions (IRFs) of the SAR system sharper and reduces interference among backscattered signals from the scatterers. However, the spatial resolution of satellite SAR is predetermined by the development requirements and design process of the satellite SAR system, considering the operational objectives and application field of the corresponding SAR mission. Therefore, it cannot be adjusted by the users.

To overcome this limitation, super-resolution (SR) methods have been proposed to improve the spatial resolution of target images for SAR and inverse SAR (ISAR) [9,10,11,12,13,14,15,16]. The SR methods in [9,10,11,12,13,14,15,16] are mostly based on spectral estimation (SPE) techniques such as multiple signal classification (MUSIC), estimation of signal parameters through rotational invariance techniques (ESPRIT), relaxation (RELAX), and autoregressive (AR) model-based linear prediction (LP). In [17], the compressive sensing (CS) technique was also used to generate super-resolved target images by solving optimization problems based on the radar signal model. The above SR methods all utilized complex radar signals, because phase information is very important to process radar images.

In [18,19,20], CS theory was utilized to conduct learning-based SR for SAR and optical images. The primary idea of learning-based SR is the learning correspondence between low-resolution (LR) and high-resolution (HR) image patches from the training database. In [18], the concept of multi-dictionary CS was proposed to jointly train low- and high-resolution dictionaries, generating super-resolved SAR patches. However, the training process may be time-consuming, which is not appropriate for real-time SAR applications. In addition, the method in [18] uses only amplitude information to make a feature vector from a SAR patch. In this case, the principal information in SAR images may be lost. In [19,20], some learning-based SR strategies were presented to enhance spatial resolutions of optical images. The method in [19] extracted similar image patches existing in the same LR remote sensing image, which was called structural self-similarity (SSSIM). Then, pre-HR images obtained by applying an interpolation process to SSSIM were utilized for dictionary training based on K-singular value decomposition (K-SVD). In [20], a blurring matrix is introduced in order to enhance the incoherency between the sparsifying dictionary and the sensing matrices. In addition, the method in [21] proposed an image deblurring method using derivative CS when accurate knowledge of the blurring operator is lacking. In [22], a CS model-based reconstruction method for multi-detector signal acquisition was presented.

It should be noted that a target observed by SAR can be represented by a combination of the target’s scattering information and the IRF of the SAR system; the IRF generally has a sinc-like shape. Naturally, it is desirable that the super-resolved image is also a combination of the target’s scattering information and the sinc-like IRF with improved spatial resolution. However, most of the above SR techniques are limited in terms of their ability to retain the sinc-like IRF in the super-resolved image. The MUSIC method computes the spatial spectral function using predefined direction vectors and the noise subspace of the target image to create a super-resolved image, after which the sinc-like shape of the IRF is completely lost. Additionally, the ESPRIT and RELAX methods estimate the geometric locations (GLs) and radar cross-sections (RCSs) of the main scatterers (i.e., line spectra), leading to multiple points in the resulting image that cannot contain the sinc-like shape of the IRF. In [23], AR model-based LP and CS techniques were used for the SR procedure of satellite SAR images to retain the sinc-like shape of the IRF well in the super-resolved image; it was demonstrated that the methods in [23] could maintain the sinc-like shape of the IRF well at a low degree of SR, yielding reliable SR performances.

However, as the degree of SR increases, the SR performances of AR model-based LP and CS techniques may degrade. In the case of AR model-based LP, the extrapolation errors may grow significantly as the degree of SR increases. This is because a simple AR model cannot effectively handle complex combinations of radar signals from many distributed scatterers in the target response at a high degree of SR. In addition, in the case of CS techniques, a high degree of SR induces only multiple point-like information in 2D images, resulting in the severe destruction of the sinc-like characteristics of the IRF in the resulting image. Consequently, it is difficult to generate reliable super-resolved results with a high degree of SR.

To overcome this problem, we propose an efficient SR method for targets observed using satellite SAR images. In short, we conceptually combine two factors: (1) the GLs and RCSs of dominant scattering centers (SCs) in the target image, and (2) the IRF of the satellite SAR system (IRF-S). First, a small target image extracted from a large-scale SAR image is subjected to proper signal processing. The principal scatterers of the targets are then extracted from the target image using the CS technique. Secondly, a SAR image of a corner reflector (CR) extracted from a large-scale satellite SAR image undergoes clutter signal removal and normalization, generating an IRF-S. Subsequently, the spatial resolution of the IRF-S is improved using AR model-based LP. Finally, the super-resolved IRF-S is convolved with the extracted SCs to generate a clear super-resolved target image. In this study, we used Korea Multi-Purpose SATellite-5 (KOMPSAT-5, K-5) images obtained at a high frequency (*X*-band) to analyze the SR performance of the proposed method.

The greatest advantage of the proposed method is that it only requires the improvement of the spatial resolution of the IRF-S containing ideal point target information to generate the super-resolved image, instead of considering a large number of scatterers; this can assist in reducing the extrapolation error of AR model-based LP at a high degree of SR. Consequently, the proposed method efficiently produces reliable super-resolved target images with a high degree of SR, even though the target response has a complex spatial distribution of scatterers.

The major objectives of this study can be summarized as follows. The first objective is to practically design an efficient SAR SR scheme that has robustness at a high SR degree. It should be noted that the proposed SR scheme contains proper preprocessing steps to cope with various types of motions of targets occurring in real situations; this can assist in improving the applicability of the proposed scheme to real systems. The second objective is to effectively verify the SR capabilities of the proposed scheme. In our experiments, various metrics such as peak signal-to-noise ratio (PSNR), structural similarity index (SSIM), focus quality parameters, and computation time (CT) as well as qualitative analysis are used to demonstrate the effectiveness of the proposed SR scheme.

## 2. Proposed SR Method for Target Image

### 2.1. Overall Flowchart of the Proposed Method

**Figure 1:**
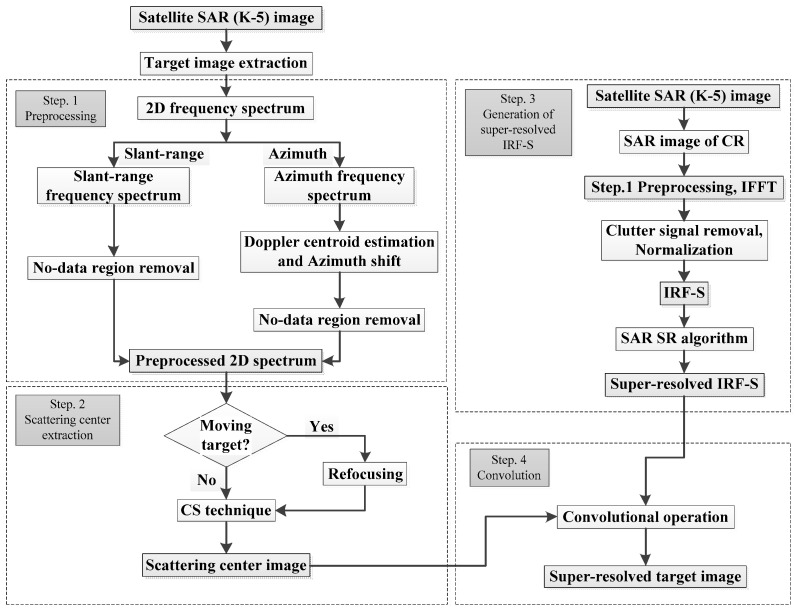
Overall flowchart of the proposed method, which consists of four steps: (1) preprocessing, (2) SC extraction, (3) generation of the super-resolved IRF-S, (4) convolution of the SC image and super-resolved IRF-S.

Figure 1 shows the overall flowchart of the proposed method, which consists of four steps: (1) preprocessing, (2) SC extraction (SCE), (3) generation of the super-resolved IRF-S, and (4) convolution of the SC image and super-resolved IRF-S. Notably, the target image can be extracted from a large-scale K-5 image through manual inspection or using various target detection algorithms [1,2,3,4]. These four steps are described in detail in the following section.

### 2.2. Radar Signal Model for the Proposed Method

According to the high-frequency scattering theory, a backscattered field in the high-frequency region can be represented as the sum of fields from a discrete set of independent scattering centers (SCs) on a target [24]. For simplicity, we adopt an undamped exponential model without angle dependence and the frequency-dependence term included in the geometrical theory of diffraction (GTD) model. Then, the scattered field signals from I SCs at different frequencies f and look angles ϕ can be modeled as [25]:

(1) $$s\left(\phi ,f\right)=\sum_{i=1}^{I}a_{i}\exp\left(-j2k\sin\phi \cdot y_{i}\right)\exp\left(-j2k\cos\phi \cdot x_{i}\right)$$

where $a_{i}$ represents the amplitude of the i-th SC at $\left(x_{i},y_{i}\right)$ and k=2πf/c denotes the wavenumber. Let $f_{x}=f\cos\phi$ and $f_{y}=f\sin\phi$. Then, Equation (1) can be expressed as follows:

(2) $$s\left(n_{az},n_{sl}\right)=\sum_{i=1}^{I}a_{i}\exp\left(-j2\pi n_{az}\cdot \frac{y_{i}}{R_{y}}\right)\exp\left(-j2\pi n_{sl}\cdot \frac{x_{i}}{R_{x}}\right)$$

where $R_{y}=c/(2\Delta f_{y})$ and $R_{x}=c/(2\Delta f_{x})$ represent the maximum unambiguous ranges in the azimuth and slant-range directions, respectively. $n_{az}$ and $n_{sl}$ denote azimuth and slant-range frequencies, respectively. p and q denote the azimuth and slant-range frequency indices, respectively. If the 2D target image domain is discretized by a 2D P×Q grid, Equation (2) can be expressed as follows:

(3) $$s\left(n_{az},n_{sl}\right)=\sum_{p=0}^{P-1}\sum_{q=0}^{Q-1}a_{p,q}\exp\left(-j\frac{2\pi }{P}n_{az}p\right)\exp\left(-j\frac{2\pi }{Q}n_{sl}q\right)$$

### 2.3. Preprocessing (Step 1)

**Figure 2:**
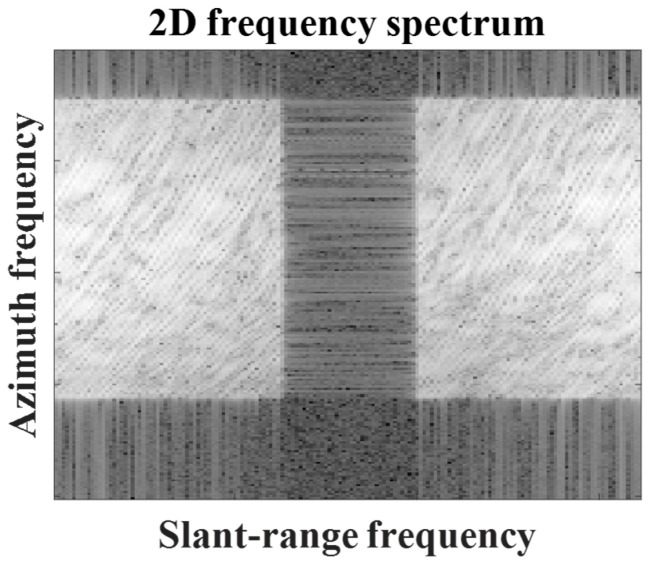
2D frequency spectrum of target image.

In Step 1, the original target image is transformed to be appropriate for the subsequent steps of the proposed SR method. First, the small target image is decompressed using a 2D fast Fourier transform (FFT) along the slant-range and azimuth directions, yielding the 2D frequency spectrum shown in Figure 2. The frequency spectrum contains no-data regions induced by oversampling in the SAR processor (SARP) along the slant-range and azimuth directions (black regions in Figure 2) [23,26].

The no-data regions break the continuity of the target information in the 2D frequency spectrum, thereby impeding successful SR processing. Thus, it is desirable to remove the no-data regions from the 2D frequency spectrum. In the case of the slant-range direction, no-data regions are always found in the middle part of the spectra owing to the characteristics of SAR processing. Therefore, no-data regions can be directly removed using the metadata provided by the SARP. Meanwhile, in the azimuthal direction, no-data regions are located in the vicinity of the Doppler centroid. Thus, the Doppler centroid is estimated, and no-data regions are removed using metadata. In this study, we refer to the preprocessed (PR) 2D frequency spectrum whose no-data regions are removed as $s^{\prime }(n_{az},n_{sl})$.

(4) $$s\prime \left(n_{az},n_{sl}\right)=\sum_{p=0}^{P-1}\sum_{q=0}^{Q-1}a_{p,q}\exp\left(-j\frac{2\pi }{P}n_{az}p\right)\exp\left(-j\frac{2\pi }{Q}n_{sl}q\right)$$

where $n_{az}=1,\;2,\;\ldots ,\;N_{az}$, $n_{sl}=1,\;2,\;\ldots ,\;N_{sl}$, and $N_{az}$ and $N_{sl}$ denote the numbers of pixels in the azimuth and slant-range frequency directions, respectively. As Equation (4) is a well-known FT relationship, it can be rewritten as the following matrix equation:

(5) $${\left[S\prime \right]}_{N_{az}\times N_{sl}}={\left[F_{az}\right]}_{N_{az}\times P}{\left[A\right]}_{P\times Q}{\left[F_{sl}^{T}\right]}_{Q\times N_{sl}}$$

where $S\prime =s\prime \left(n_{az},n_{sl}\right)$ denotes the $N_{az}\times N_{sl}$ matrix, $A=a_{p,q}$ denotes the P×Q matrix, $F_{az}$ denotes the $N_{az}\times P$ Fourier dictionary in the azimuthal direction, and $F_{sl}$ denotes the $Q\times N_{sl}$ Fourier dictionary in the slant-range direction.

### 2.4. Scattering Center Extraction (Step 2)

In radar signal processing, SCE can be effectively accomplished using various CS or SPE techniques, such as the orthogonal matching pursuit (OMP) [27], root-MUSIC [15], and ESPRIT [14] algorithms, provided that the backscattered field satisfies the signal modeling in Equation (4). Unlike SPE techniques, CS techniques avoid the need to estimate the number of SCs. This is a big advantage for SCE because the estimation of the number of SCs is very difficult for an extended target. It should be noted that the most important thing for the SCE step in the proposed scheme is the computation time (CT), because the main application of the proposed scheme is target recognition using satellite SAR images requiring real-time processing. In the area of radar imaging, the OMP algorithm, which is the most popular greedy pursuit method based on the CS technique, has provided reliable accuracy with very low CTs [27,28]. In addition, when we conducted experiments for SCE using several CS algorithms (OMP, MP, Lasso, BP, and BPDN), OMP exhibited the most reliable SCE performances in terms of accuracy and CT. Thus, the OMP algorithm was adopted for SCE in this study.

In the case of the stationary target (ST), its PR 2D frequency spectrum, $s_{ST}^{\prime }\left(n_{az},n_{sl}\right)$, can be well matched with Equation (4). After we assume $P>N_{az}$ and $Q>N_{sl}$, the SC image can be obtained by solving the $l_{0}$-norm minimization problem as follows:

(6) $$(P_{0}):\;\;\underset{A}{\min}{\left\|A\right\|}_{0}\;\mathrm{subject}\;\mathrm{to}\;S_{ST}^{\prime }=F_{az}AF_{sl}^{T}$$

where $S_{ST}^{\prime }=s_{ST}^{\prime }\left(n_{az},n_{sl}\right)$ denotes the $N_{az}\times N_{sl}$ matrix. Because the optimization of the nonconvex $\left(P_{0}\right)$ is an NP-hard problem that is extremely complex and difficult to solve, the OMP algorithm suboptimally selects the best solution at every iteration until the convergence criterion is satisfied [27].

**Figure 3:**
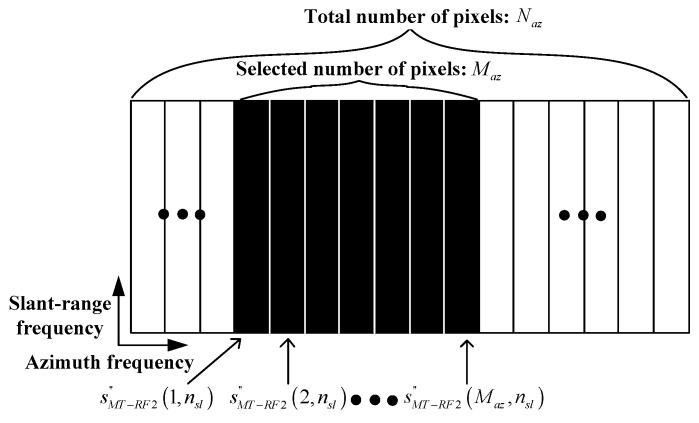
ROFOC 2D frequency spectrum for moving target with complex 3D motion.

Meanwhile, the PR 2D frequency spectrum of the moving target (MT), $s_{MT}^{\prime }\left(n_{az},n_{sl}\right)$, differs significantly from Equation (4) due to the target’s motion-induced phase, which leads to severe blurring of the target response in the target image. Therefore, in this study, the refocusing technique is applied to $s_{MT}^{\prime }\left(n_{az},n_{sl}\right)$ if the corresponding target image contains the blurred target response of the moving target [29,30]. The refocusing technique can be carried out in two different ways: (1) only phase adjustment (PA) and (2) PA with optimal time windowing (OTW). In the case of moderate target motion, it is enough to use only the PA algorithm to obtain a refocused (REFOC) 2D frequency spectrum $s_{MT,\;RF1}^{\prime \prime }\left(n_{az},n_{sl}\right)$; $n_{az}=1,\;2,\;\ldots ,\;N_{az}$, $n_{sl}=1,\;2,\;\ldots ,\;N_{sl}$, whose signals are well-matched with Equation (4). A clear target response can be obtained by applying the IFFT processing to $s_{MT,\;RF1}^{\prime \prime }\left(n_{az},n_{sl}\right)$. Furthermore, the number of pixels in $s_{MT,\;RF1}^{\prime \prime }\left(n_{az},n_{sl}\right)$ is the same as that of $s_{MT}^{\prime }\left(n_{az},n_{sl}\right)$. Meanwhile, if a target has 3D dynamic motion, its effective rotation vector (ERV) varies during the coherent processing interval (CPI). For example, a moving ship can have complex 3D self-motion, such as roll, pitch, and yaw due to waves and offshore winds. In this case, only using the PA algorithm cannot solve the mismatch between $s_{MT}^{\prime }\left(n_{az},n_{sl}\right)$ and Equation (4). OTW [31] selects an optimal time window in which the ERV of the ship is nearly constant. Thus, the combination of OTW and the PA algorithm can effectively cope with the target’s complex motion, yielding $s_{MT,\;RF2}^{\prime \prime }\left(n_{az},n_{sl}\right)$; $n_{az}=1,\;2,\;\ldots ,\;M_{az}$, $n_{sl}=1,\;2,\;\ldots ,\;N_{sl}$. Notably, the number of pixels in $s_{MT,\;RF2}^{\prime \prime }\left(n_{az},n_{sl}\right)$ is smaller than that in $s_{MT}^{\prime }\left(n_{az},n_{sl}\right)$ along the azimuthal direction (i.e., $M_{az}<N_{az}$) in general, because OTW only selects a certain part of the total signals collected in the CPI, as shown in Figure 3. This implies that the azimuth frequency bandwidth of $s_{MT,\;RF2}^{\prime \prime }\left(n_{az},n_{sl}\right)$ is smaller than that of $s_{MT}^{\prime }\left(n_{az},n_{sl}\right)$. After the refocusing technique has been applied to $s_{MT}^{\prime }\left(n_{az},n_{sl}\right)$, the SCs are extracted using the same method as that used for $s_{ST}^{\prime }\left(n_{az},n_{sl}\right)$:

(7) $$(P_{0}):\;\;\underset{A}{\min}{\left\|A\right\|}_{0}\;\mathrm{subject}\;\mathrm{to}\;S_{MT}^{\prime }=F_{az}AF_{sl}^{T}$$

where $S_{MT}^{\prime }$ denotes the $S_{MT,RF1}^{\prime }$ or $S_{MT,RF2}^{\prime }$, $S_{MT,RF1}^{\prime }=s_{MT,RF1}^{\prime }\left(n_{az},n_{sl}\right)$ is the $N_{az}\times N_{sl}$ matrix, and $S_{MT,RF2}^{\prime }=s_{MT,RF2}^{\prime }\left(n_{az},n_{sl}\right)$ is the $M_{az}\times N_{sl}$ matrix.

### 2.5. Generation of Super-Resolved IRF-S (Step 3)

In SAR signal processing, a target observed by satellite SAR can be represented by a combination of the SC image containing the target’s scattering information (i.e., geometric locations and radar cross-sections) and 2D IRF-S, as follows [26,32]:

(8) $$TI\left(p,q\right)=\sum_{i=1}^{I}r_{i}f_{2D}\left(p-p_{i},q-q_{i}\right)$$

where $f_{2D}\left(p-p_{i},q-q_{i}\right)=f_{az}\left(p-p_{i}\right)f_{sl}\left(q-q_{i}\right)$ denotes the 2D IRF-S shifted by $p_{i}$ and $q_{i}$, respectively; $f_{az}\left(p-p_{i}\right)$ and $f_{sl}\left(q-q_{i}\right)$ denote the 1D IRF-S along the azimuthal and slant-range directions shifted by $p_{i}$ and $q_{i}$, respectively; $r_{i}$ denotes the RCS of the *i*-th scatterer of the target; and $p_{i}$ and $q_{i}$ denote the target’s position along the azimuth and slant-range directions, respectively. In Equation (8), it is assumed that the same 2D IRF-S is combined with all the scatterers of the target because the scatterers of the target are generally concentrated in a small area. Because the SC image already has all the geometric locations and radar cross-sections of the target’s scatterers (i.e., $p_{i}$, $q_{i}$, and $r_{i}$), Equation (8) can be reformulated in the 2D image domain as follows:

(9) $$TI\left(p,q\right)=I_{SC}⊗f_{2D}(p,q)$$

where $I_{SC}$ denotes the SC image, ⊗ denotes the convolutional operation, and $f_{2D}\left(p,q\right)$ denotes the 2D IRF-S. Thus, only the 2D IRF-S $f_{2D}\left(p,q\right)$ is required.

To obtain the 2D IRF-S, it is desirable to use a SAR image of an isolated point target. This is because it can wholly represent the quality parameters of the satellite SAR system such as the 3-dB bandwidth (i.e., spatial resolution), peak side–lobe ratio (PSLR), and integrated side–lobe ratio (ISLR), without interference from other scatterers. In this study, a SAR image of a CR was first extracted from a large-scale satellite SAR image. Next, the preprocessing step in Step 1 was applied to the SAR image of the CR to remove the no-data region in the frequency spectrum, followed by IFFT processing, yielding a PR image of the CR. However, the PR image of the CR cannot be directly regarded as the IRF-S because it contains many clutter signals reflected from the background and is amplified by the RCS of the target. To solve this problem, slant-range and azimuth cuts were obtained by cutting the PR image of the CR at the center pixels in the slant-range and azimuth directions, respectively. Then, the multiplication of the slant-range and azimuth cuts resulted in a clean PR image of the CR, where the clutter signals were almost completely removed. Subsequently, the clean PR image was normalized by the maximum amplitude, yielding a 2D IRF-S $f_{2D}\left(p,q\right)$.

Subsequently, the spatial resolution of the 2D IRF-S was improved using a conventional SAR SR algorithm based on AR model-based LP [16,17]. In this study, the Burg algorithm was adopted because of its efficiency with respect to accuracy and complexity (CT). The Burg algorithm extends the frequency bandwidths of scattered field signals through extrapolation and generates a new image with improved spatial resolution. Let 1D scattered field signals along the azimuth frequency or slant-range frequency direction at a specific slant-range or azimuth bin be noted by $s_{1D}(n)$; n=1,2, …, N, where N is either $N_{az}$ (in the case of the AR model in the azimuthal frequency direction) or $N_{sl}$ (in the case of the AR model in the slant-range frequency direction). The Burg algorithm utilizes the AR model, which assumes that $s_{1D}(n)$ is the sum of the undamped exponentials [11,16,17]. In the AR model, $s_{1D}(n)$ must satisfy the following forward and backward linear prediction conditions:

(10) $${\hat{s}}_{1D}(n)=\left\{\begin{array}{l} -\sum_{i=1}^{k}\gamma_{i}s_{1D}(n-i),\;\;\;\;\;n=k+1,\;k+2,\;\ldots ,\;N\\ -\sum_{i=1}^{k}\gamma_{i}^{*}s_{1D}(n+i),\;\;\;\;\;n=1,\;2,\;\ldots ,\;N-k\;\;\;\;\;\;\; \end{array}\right.$$

where * denotes the complex conjugate, $\gamma_{i}$ denotes the coefficients of the AR model, k is the AR model order, and ${\hat{s}}_{n}$ is the estimated data using forward or backward prediction. The forward prediction error $e_{n}^{f}$ and backward prediction error $e_{n}^{b}$ can be defined as follows:

(11) $$e_{n}^{f}={\left|s_{1D}(n)-{\hat{s}}_{1D}(n)\right|}^{2}={\left|\sum_{i=0}^{k}\gamma_{i}s_{1D}(n-i)\right|}^{2},\;\;n=k+1,\;k+2,\;\ldots ,\;N$$

(12) $$e_{n}^{b}={\left|s_{1D}(n)-{\hat{s}}_{1D}(n)\right|}^{2}={\left|\sum_{i=0}^{k}\gamma_{i}^{*}s_{1D}(n+i)\right|}^{2},\;\;n=1,\;2,\;\ldots ,\;N-k$$

where $\gamma_{0}=1$. To minimize the sum of forward and backward prediction errors in Equations (11) and (12), the Burg method determines the coefficients of the AR model $\gamma_{i}$. In this study, we chose k=N/3 because it provides a robust estimation of $a_{i}$ [33]. After $a_{i}$ had been obtained, the number of additional cells required for extrapolation was determined as follows:

(13) $$L=\mathrm{Round}\left[N\times \left(SR_{bef}/SR_{aft}-1\right)/2\right]$$

where $\mathrm{Round}\left[\cdot \right]$ denotes the round-off operator, and $SR_{bef}$ and $SR_{aft}$ are the spatial resolutions in the azimuth or slant-range direction before and after the SR procedure, respectively. Next, L cells were added to the first and last cells of $s_{1D}(n)$. Then, the scattered field signals of the 2L cells were estimated using $\gamma_{i}$. The above extrapolation was iterated for all the azimuthal and slant-range bins. Then, 2D IFFT was applied to the total scattered field signals to generate a 2D IRF-S with improved spatial resolution, referred to as $f_{2D-IMP}\left(p,q\right)$. The Burg algorithm assumes L to be linearly proportional to the increment of the azimuth and slant-range frequency bandwidths, which are directly associated with the SAR image resolutions. Thus, the spatial resolutions of $f_{2D-IMP}\left(p,q\right)$ can be determined by controlling L.

### 2.6. Convolution of SC Image and Super-Resolved IRF-S (Step 4)

As the last step in generating the super-resolved target image, $f_{2D-IMP}\left(p,q\right)$ in Section 2.5 is convolved with $I_{SC}$ in Section 2.4 according to Equation (9).

## 3. Experimental Results

To investigate the effectiveness of the proposed method, we considered ship targets observed by K-5. We extracted each target image from a large-scale K-5 image. Additionally, we extracted CR images from other large-scale K-5 images of a real CR located at the KOMPSAT calibration site in Mongolia. Notably, the target and CR images were obtained using the same observation mode (spotlight), beam number, and polarization (HH). In this study, we analyzed SR performance from two perspectives: (1) restoration and (2) improvement.

In many studies on the development of SR algorithms, restoration metrics have been widely used to evaluate SR capability. In this study, we used two restoration metrics: the peak signal-to-noise ratio (PSNR) and structural similarity index (SSIM), which have been used in optical image-based SR algorithms [34,35]. Let a PR and REFOC (optional depending on the motion of the ship) target image be referred to as the reference (REF) target image. When PSNR and SSIM were used to evaluate the SR capability, the spatial resolution of the REF target image was intentionally worsened by reducing the slant-range and azimuth frequency bandwidth, yielding an LR target image. Similarly, the spatial resolution of the IRF-S is degraded, leading to an LR IRF-S. The proposed SR method was then applied to the LR target image and the LR IRF-S to generate a restored target image whose spatial resolution was the same as that of the REF target image. The PSNR and SSIM compute the similarity of the scattering information between two images (i.e., the REF target image and the restored target image) to evaluate the SR capability more accurately than the focus qualities. The PSNR is the ratio between the maximum signal and the corrupting noise that affects high-resolution reconstruction:

(14) $$PSNR=20\log_{10}(MAX_{I})-10\log_{10}MSE(x,y)$$

where $MAX_{I}$ denotes the maximum possible pixel value of the image, and MSE(x,y) denotes the mean squared error between the two images, x and y. The SSIM is a metric used to evaluate the similarity between two images by combining brightness, contrast, and structural information.

(15) $$SSIM(x,y)=\frac{\left(2\mu_{x}\mu_{y}+c_{1}\right)\left(2\sigma_{xy}+c_{2}\right)}{\left(u_{x}^{2}+\mu_{y}^{2}+c_{1}\right)\left(\sigma_{x}^{2}\sigma_{y}^{2}+c_{2}\right)}$$

where $\mu_{x}$, $\mu_{y}$, $\sigma_{x}$, $\sigma_{y}$, and $\sigma_{xy}$ denote the local means, standard deviations, and cross-covariances of x and y, respectively. $c_{1}$ and $c_{2}$ are small constants. Generally, higher PSNR and SSIM indicate better restoration performance and vice versa.

### 3.1. SR Results for Static Ship Target

**Figure 4:**
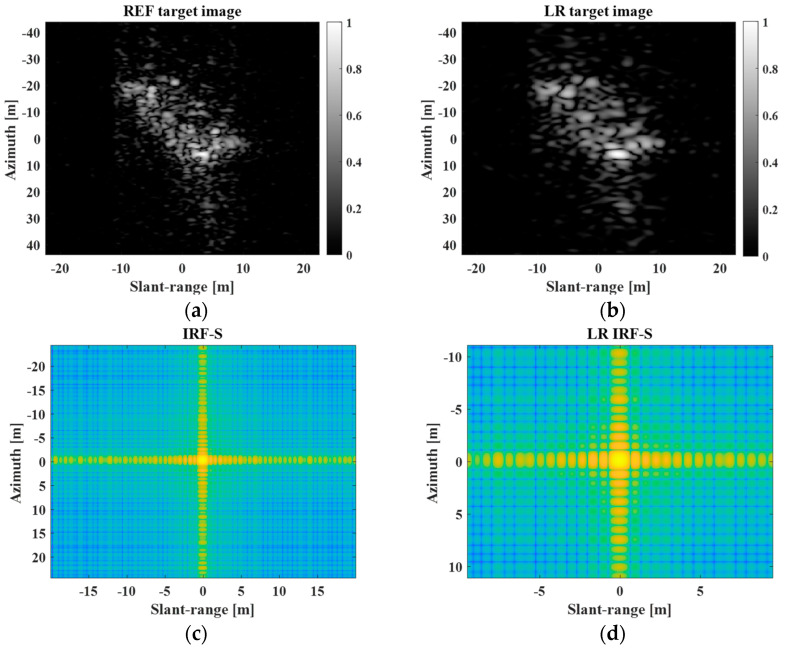
REF and LR target images for stationary ship target with PR and LR IRF−Ss. (**a**) REF target image, (**b**) LR target image, (**c**) PR IRF−S, (**d**) LR IRF−S.

The ratio of the adjusted spatial resolution to the original spatial resolution is denoted as d. Figure 4 shows the REF and LR target images for the stationary ship target with PR and LR IRF-Ss when d=2.

**Figure 5:**
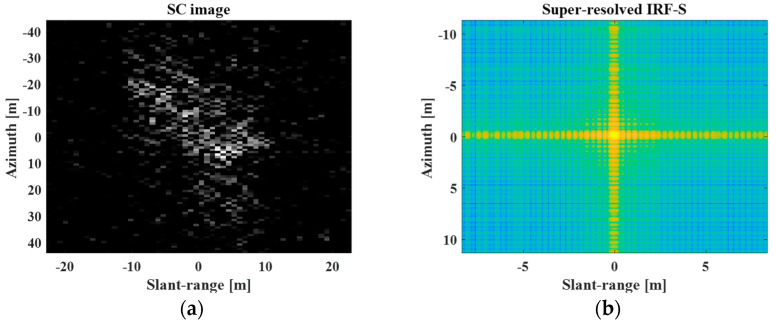
SC image and super−resolved IRF−S. (**a**) SC image, (**b**) super-resolved IRF−S.

In Figure 4b,d, the spatial resolutions of the REF target image and PR IRF-S are degraded by reducing the frequency bandwidth along the slant-range and azimuthal directions. Next, Figure 5 shows the SC image and super-resolved IRF-S obtained using the processing steps described in Section 2.4 and Section 2.5, respectively. In Figure 5, the SC image shows the primary scattering information for the target response. Additionally, the super-resolved IRF-S generated from the LR IRF-S was similar to the PR IRF-S.

**Figure 6:**
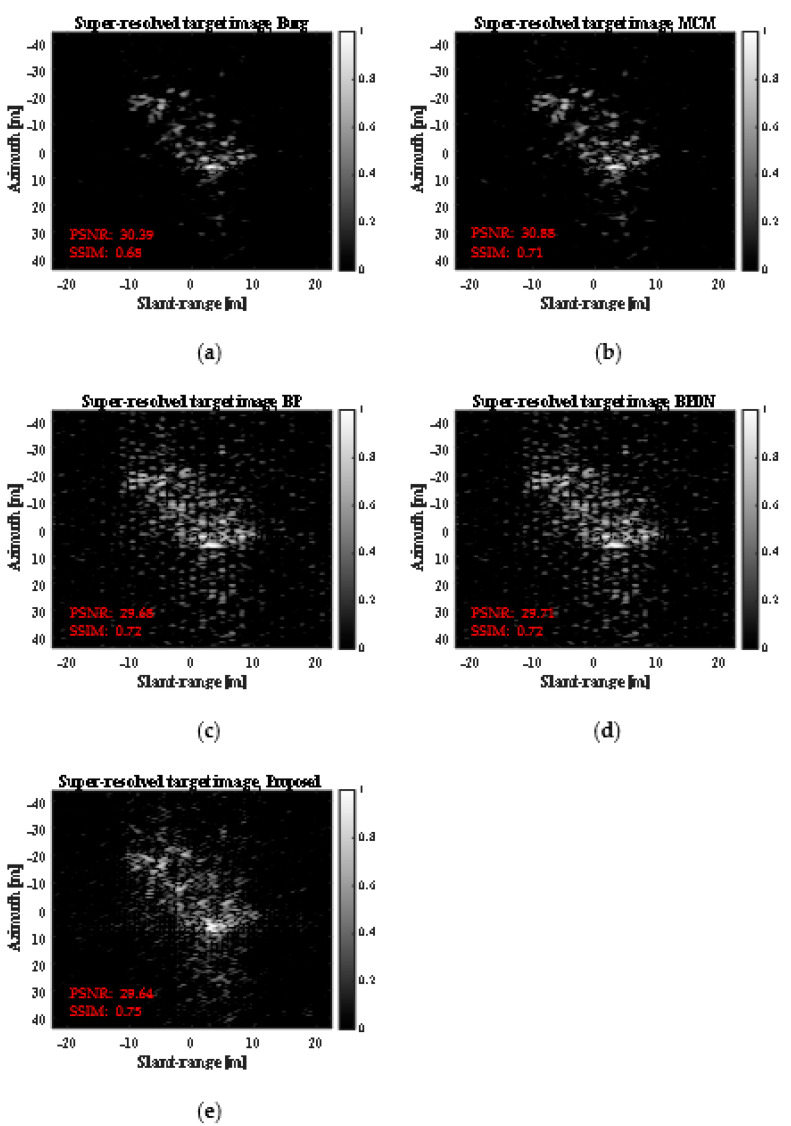
SR results obtained using five SR methods for static ship target. (**a**) Burg, (**b**) MCM, (**c**) BP, (**d**) BPDN, (**e**) proposed.

Figure 6 shows the SR results obtained using the five SR methods (the four SR methods in [23], and the proposed SR method). As shown in Figure 6, the five SR methods generated slightly different SR results, all of which were similar to the REF target image shown in Figure 4. Evidently, the results from Figure 6 show a better spatial resolution than the LR target image in Figure 4b. However, when observed with the naked eye, it is difficult to determine which algorithm has a better SR capability.

To conduct a quantitative analysis of the SR capabilities of the five SR methods, the PSNRs and SSIMs were computed by varying d from 2 to 4 in increments of 0.5, as summarized in Table 1 and Table 2. In our study, the BPDN algorithm induces slightly different results for the same target image at every iteration, because the constraint relaxation parameter needed for the log-barrier algorithm is a random matrix. Thus, in the case of the BPDN algorithm, the results in Table 1 and Table 2 are obtained from the average values of 50 independent realizations to provide reliable performance evaluations.

In Table 1, the MCM method shows better PSNR only at d=2. Otherwise, the PSNRs of the proposed method are the highest for all other values of d=2.5, 3, 3.5, 4. Additionally, the proposed method shows the best SSIMs over the entire range of d in Table 2.

In addition, Table 3 shows the standard deviations of the BPDN algorithm for the results in Table 1 and Table 2.

In Table 3, the standard deviations of BPDN are almost all very small values lower than 0.0001. Thus, the BPDN algorithm is very statistically stable for solving our problem.

### 3.2. SR Results for Moving Ship Target—Moderate Motion

**Figure 7:**
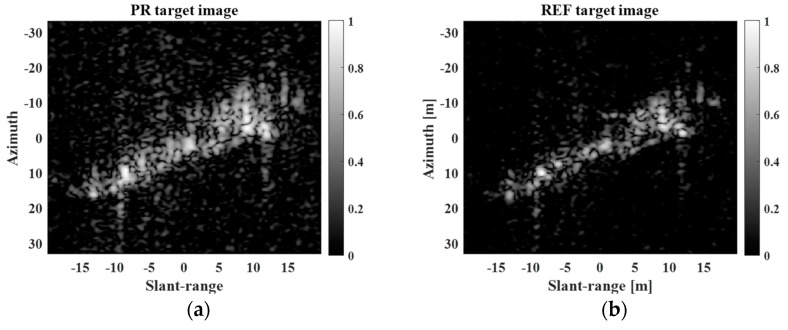
PR and REF target images for moving ship target with moderate motion. (**a**) PR target image, (**b**) REF target image.

In this section, we consider another ship target with moderate motion. In this case, although the target response in Figure 7a is blurred owing to the motion of the target, its phase errors can be effectively eliminated using only the PA algorithm. In this study, the entropy minimization method in [36] was selected as the PA algorithm. Figure 7 shows the PR and REF target images of a moving target with moderate motion. As shown in Figure 7b, the PA algorithm successfully removes the blurring effect of the target response resulting from motion-induced phase errors.

**Figure 8:**
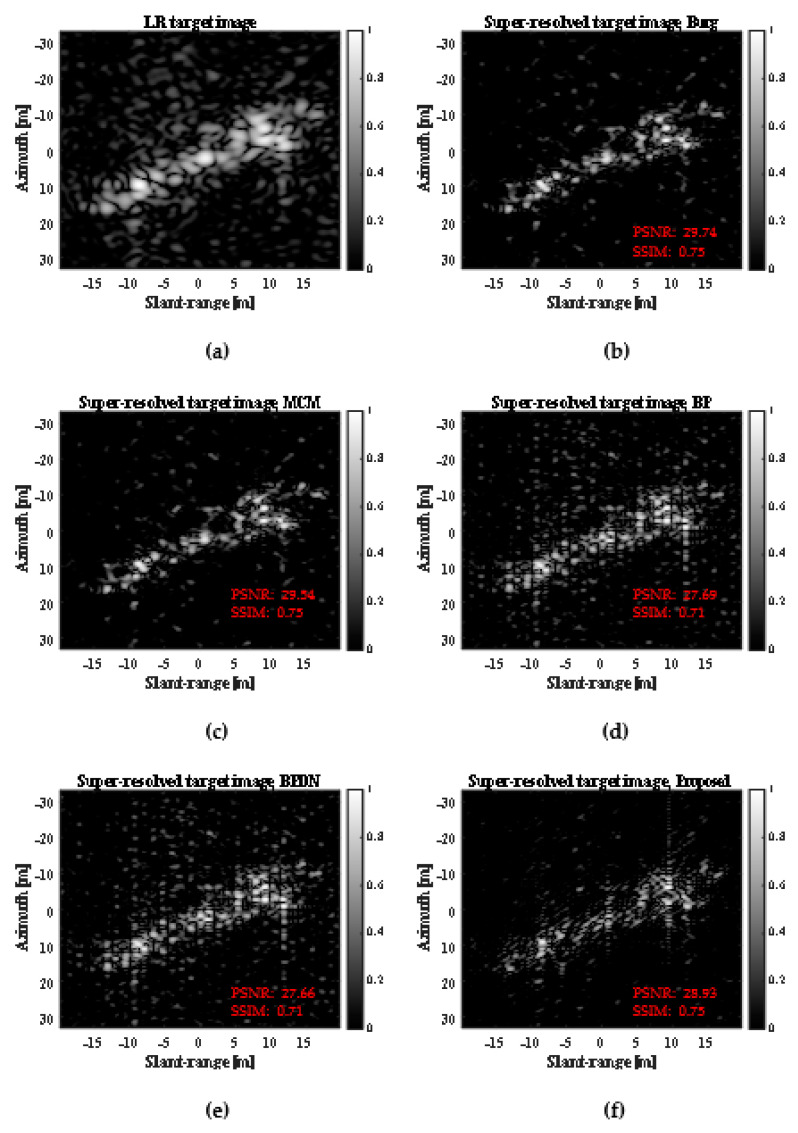
LR target image and SR results obtained using five SR methods for moving ship target with moderate motion. (**a**) LR target image, (**b**) Burg, (**c**) MCM, (**d**) BP, (**e**) BPDN, (**f**) proposed.

Figure 8 shows the LR target image for a moving ship target with moderate motion and the corresponding SR results obtained using the five SR methods when d=2.

In Figure 8, all the five methods enhance the spatial resolution of the LR target image, generating different super-resolved scattering information results, which are all similar to the REF image in Figure 7b. Table 4 and Table 5 show the PSNRs and SSIMs for super-resolved images in Figure 8, respectively. In Table 4 and Table 5, the results of the BPDN algorithm were obtained from the average values of 50 independent realizations to provide reliable performance evaluations. In the case of PSNR, the Burg method shows the highest performances at d=2, 2.5. However, the proposed method achieves the best PSNRs at d=3, 3.5, 4. In the case of SSIM, the proposed method shows outstanding performances at all ds, as compared with other SR methods.

### 3.3. SR Results for Moving Ship Target—Complex Motion

**Figure 9:**
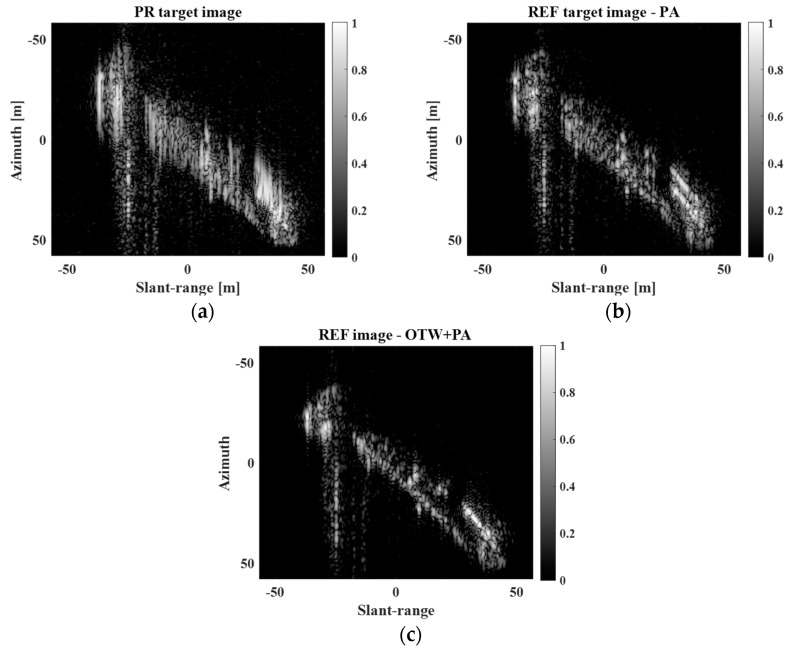
PR and REF target images for moving ship target with complex motion. (**a**) PR target image, (**b**) REF target image−PA, (**c**) REF target image−OTW + PA.

As mentioned in Section 2, a ship target can have 3D complex motion due to waves and offshore wind. In this case, the ERV varies during the CPI, resulting in a severely degraded target response. Thus, using only the PA algorithm has limitations in dealing with the blurring effect of the target response, leading to a mismatch between $s_{MT}^{\prime }\left(n_{az},n_{sl}\right)$ and Equation (4). Figure 9 shows the PR image, REF image obtained using only the PA algorithm [36], and REF image obtained using both the OTW [29] and PA [36] algorithms for moving ship targets with complex motion. Notably, as shown in Figure 9b, the target response still contains many phase errors, which cause blurring of the target response. This is because the PA algorithm cannot handle the 3D motion components of the target. The combination of the OTW and PA algorithms can generate a clear target response, as shown in Figure 9c. Thus, it is necessary to utilize both the OTW and PA algorithms to obtain the correct scattering information for a moving target with complex motion.

**Figure 10:**
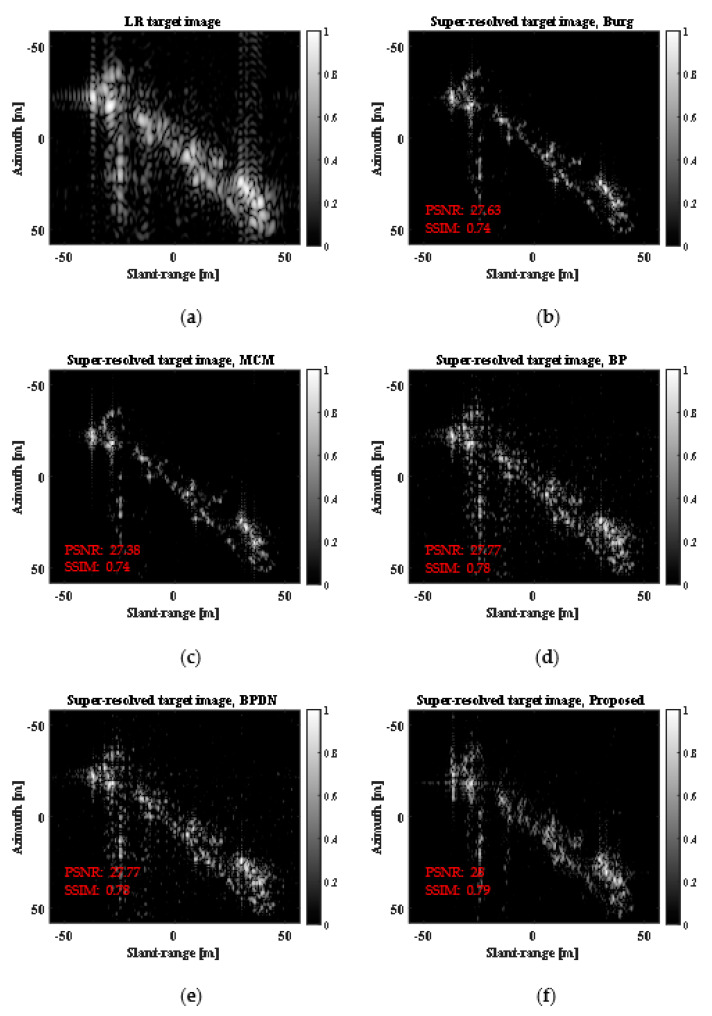
LR target image and SR results obtained using five SR methods for moving ship target with complex motion. (**a**) LR target image, (**b**) Burg, (**c**) MCM, (**d**) BP, (**e**) BPDN, (**f**) proposed.

Figure 10 shows the LR target image and the corresponding SR results obtained using the five SR methods for moving targets with complex motion when d=2.5. In Figure 10a, the spatial resolution of the LR target image considerably deteriorates compared with that of the REF image in Figure 9c. In the case of the super-resolved images, the five algorithms led to slightly different scattering information for the target. When observed with the naked eye, it is difficult to determine which algorithm has better SR capability. Although these super-resolved images are not identical to the REF image in Figure 9c, it is evident that the five SR algorithms improved the spatial resolution of the LR image in Figure 10a.

Table 6 and Table 7 list the PSNRs and SSIMs for the super-resolved images shown in Figure 10, respectively. In Table 6 and Table 7, the results of the BPDN algorithm were obtained from the average values of 50 independent realizations to provide reliable performance evaluations. In the case of the PSNR, the proposed method achieved slightly better scores for all ds. In Table 7, the SSIMs of all the algorithms are similar at d=2. Additionally, those of the proposed method were slightly better than those of the other algorithms for all other instances of ds.

### 3.4. SR Results in the Case of Improvement

**Figure 11:**
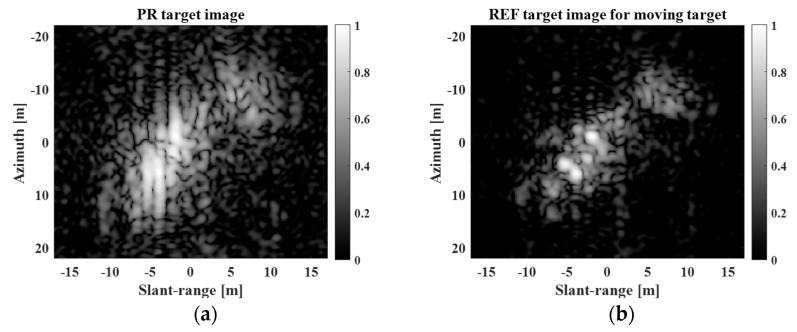
PR and REF target images for moving target with moderate motion in the case of improvement. (**a**) PR target image, (**b**) REF target image.

In the previous sections, we demonstrated that, from a restoration perspective, the proposed scheme is useful for improving the spatial resolution of target images extracted from large-scale KOMPSAT-5 images. In addition, we examine the SR capability of the proposed method from the perspective of improvement, which is closer to a real situation. Figure 11 shows the PR and REF target images of a moving target with moderate motion. The target motion causes blurring of the target response in the target image. After the PR target image had been refocused, the REF target image contained a clear target response, as shown in Figure 11b.

**Figure 12:**
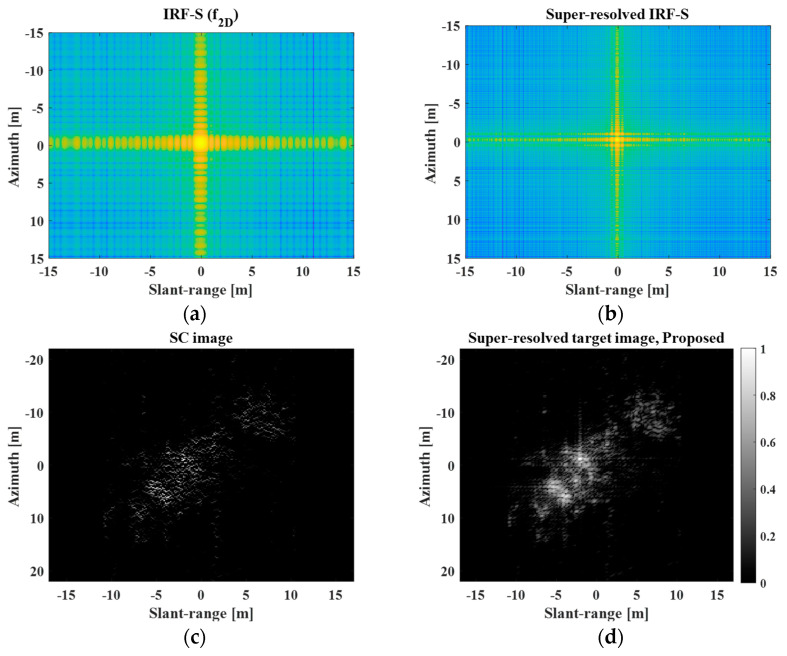
Original IRF−S, super-resolved IRF−S, SC image, and super-resolved target image. (**a**) Original IRF−S, (**b**) super-resolved IRF−S, (**c**) SC image, (**d**) super−resolved target image.

The ratio of the original spatial resolution to the adjusted spatial resolution is denoted as r. Figure 12 shows the original IRF-S, super-resolved IRF-S, SC image, and super-resolved target image obtained using the proposed scheme when r=3. From Figure 12b, it is evident that the super-resolved IRF-S has a better spatial resolution than the original IRF-S. Additionally, the SC image appeared to represent the principal scattering information of the target response, as shown in Figure 12c. Consequently, compared with Figure 11b, the proposed method significantly enhanced the spatial resolution of the target image, as shown in Figure 12d, and the IRFs of the scatterers of the target response became sharper, while the interference among the scatterers of the target response was reduced. Although measuring the degree of improvement in the spatial resolution is challenging, it is obvious that the SR results in Figure 12d provide more precise and delicate information about the principal scattering centers.

**Figure 13:**
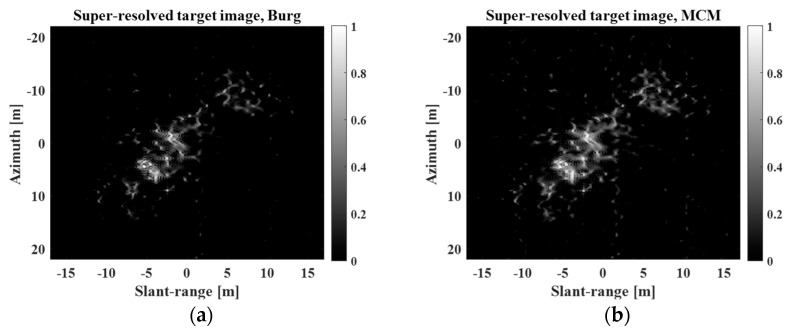

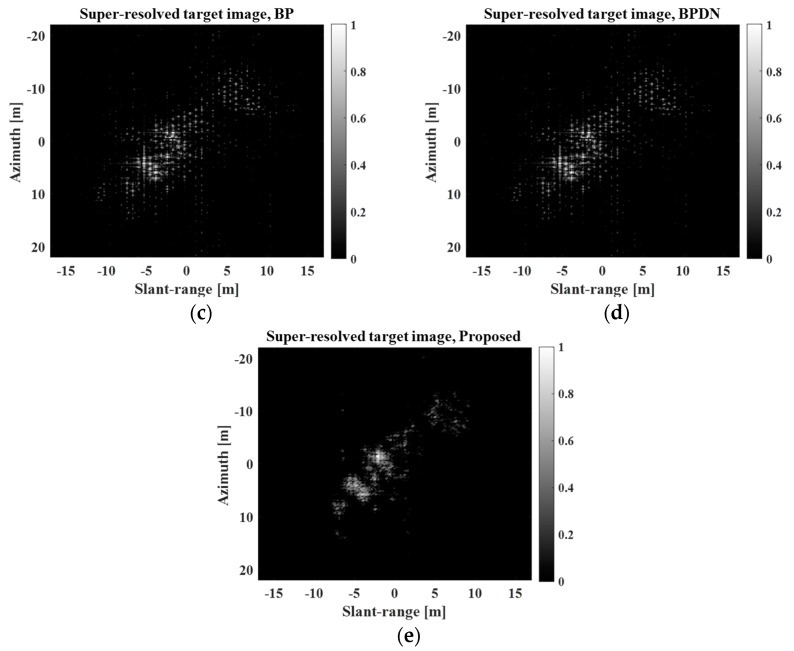
Comparison of SR results obtained using the proposed method with those obtained using the four algorithms in [23] at r=4. (**a**) Burg, (**b**) MCM, (**c**) BP, (**d**) BPDN, (**e**) proposed.

**Figure 14:**
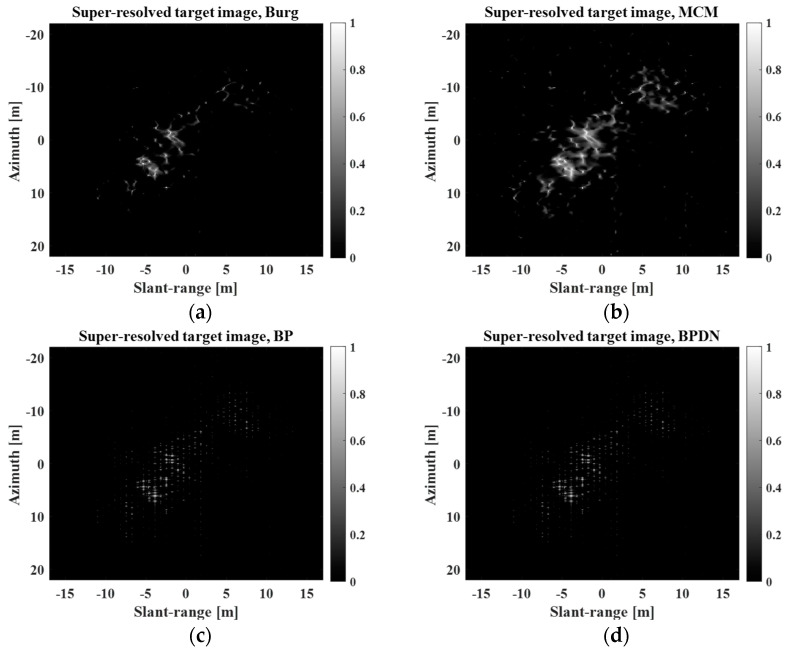

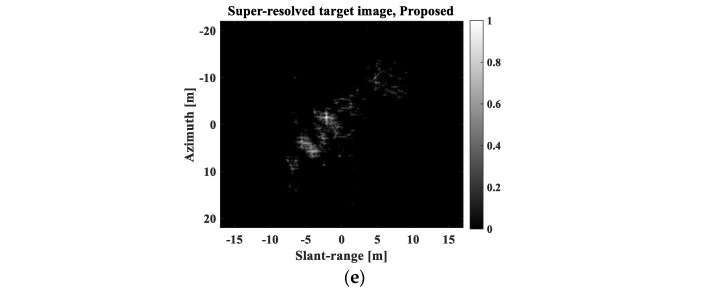
Comparison of SR results obtained using the proposed method with those obtained using the four algorithms in [23] at r=7. (**a**) Burg, (**b**) MCM, (**c**) BP, (**d**) BPDN, (**e**) proposed.

To compare the SR capability of the proposed method with that of other SR algorithms, the Burg, MCM, BP, and BPDN algorithms in [23] were also used to generate super-resolved images. Figure 13 and Figure 14 show a comparison of the SR results obtained using the proposed method with those obtained using the four algorithms (Burg, MCM, BP, and BPDN) at r=4 and r=7, respectively.

Figure 13 and Figure 14 demonstrate that all SR methods successfully improved the spatial resolution of the REF image in Figure 11b. However, the super-resolved images obtained using the four methods in [23] cannot effectively reflect the inherent sinc-like shape of the IRF. In particular, they exhibited significantly different scattering mechanisms from the REF image when there was an extremely high degree of SR (r=7). In the cases of Burg and MCM, the scattering information was largely smeared. BP and BPDN yielded multiple point-like information. Meanwhile, the proposed method can maintain the sinc-like shape of the IRF, generating a more realistic super-resolved target image regardless of the degree of SR, because it directly utilizes the super-resolved IRF-S (for example, Figure 12b) in SR processing. This indicates that the proposed method is robust for generating super-resolved images with extremely high degrees of SR.

Furthermore, the CTs of the five SR methods used for Figure 13 and Figure 14 were measured to investigate their applicability to real systems. The MATLAB program and a PC with a CPU clock speed of 3.7 GHz were used (the MATLAB program was not optimized to obtain the best computation speed).

To analyze the CTs versus r, super-resolved target images were generated by varying r from 3 to 7 in increments of 1.

Table 8 lists the CT values to generate the super-resolved target images for Figure 11b. As shown in Table 8, the Burg, MCM, and proposed methods exhibited reliable CTs, and the proposed method achieved the best CTs over the entire range of r. Considering that our equipment and software were not optimized for SR processing, the proposed method has great potential for use in real systems. Meanwhile, the CTs of the BP and BPDN methods increased exponentially as r increased. This means that BP and BPDN are not appropriate for real-time applications (e.g., SAR automatic target recognition (ATR)). In this study, we used $l_{1}$-magic [36] software by Candes and Romberg to conduct BP and BPDN. In the case of BP, linear programs are solved using a generic path-following primal-dual method. In the case of BPDN, second-order cone programs are solved with a generic log-barrier algorithm. Because BP and BPDN spend a lot of time solving the above optimization problems, they exhibit worse CTs compared with Burg, MCM, and the proposed scheme.

## 4. Discussion

In Section 3, we demonstrate that the proposed scheme improves the spatial resolution of target images extracted from large-scale KOMPSAT-5 images. This works well for various types of targets. In particular, the proposed method exhibits excellent SR performance at both high and extremely high degrees of SR (d≥2.5, r=4, 7).

In the case of restoration (i.e., Section 3.1, Section 3.2, and Section 3.3), the PSNR and SSIM were sufficient to quantitatively analyze the SR performance. However, in the case of improvement (i.e., Section 3.4), it is difficult to measure the SR capability quantitatively. In fact, we had no choice but to depend on the naked eye.

Nevertheless, we added an indirect analysis using Shannon entropy (SE) and image contrast (IC), which are widely used to evaluate the focus quality of SAR images [37,38,39]. SE and IC can be expressed as follows [37]:

(16) $$\mathrm{SE}=\sum \sum \frac{{\left|I_{2D}\right|}^{2}}{S}\cdot \ln\frac{S}{{\left|I_{2D}\right|}^{2}}$$

(17) $$\mathrm{IC}=\sigma \left[{\left|I_{2D}\right|}^{2}\right]/E\left[{\left|I_{2D}\right|}^{2}\right]$$

where $I_{2D}$ denotes a 2D image, $\sum \sum \left(\cdot \right)$ denotes the summation of all the elements in a matrix, $S=\sum \sum {\left|I_{2D}\right|}^{2}$, $E\left[\cdot \right]$ denotes the mean, and $\sigma \left[\cdot \right]$ denotes the standard deviation. Generally, a lower SE and higher IC imply a better focus quality. In addition, an improvement in focus quality can indirectly imply an improvement in the 3-dB bandwidth of the IRF and a reduction in interference among IRFs [23]. Table 9 and Table 10 list the SEs and ICs of the super-resolved images in Figure 13 and Figure 14.

In Table 9 and Table 10, the REF images exhibit better focus quality than the PR images at r=4,7. This is natural, considering the refocusing process. In addition, all super-resolved images exhibited better focus quality than the REF images at r=4,7. Particularly, the proposed method yields the best SE and IC for r=4.

The images super-resolved by BP and BPDN yield the best SEs and ICs at r=7. However, this is because the focus quality computes only the sharpness of the image instead of considering the scattering information of the target response. In fact, these methods generate only multiple points in Figure 13 and Figure 14; the sinc-like shape of the IRF is completely lost in the super-resolved image. Thus, IC and SE must be used with caution to evaluate SR performance, considering the scattering information of the resulting super-resolved images.

Furthermore, the proposed method extracts the IRF-S from real K-5 images to generate the super-resolved IRF-S; the advantage of this is that the IRF-S can be prepared in advance, because various K-5 images for the CR are already obtained for calibration and validation purposes. Thus, a super-resolved IRF-S can be generated using AR model-based LP in a short time period.

As an alternative, point-target simulation can also be used to obtain the super-resolved IRF-S. Once the REF image has been obtained, point-target simulation can generate a super-resolved IRF-S considering the frequency bandwidth of the REF target image and the degree of SR. However, point-target simulation requires complex processing to imitate the satellite SAR geometry, SAR raw signal generation, and SAR processors, resulting in long computation time. Notably, a super-resolved IRF-S cannot be prepared in advance using point-target simulation, which is a critical problem because the main application of the proposed method is SAR target recognition, which requires near-real-time processing. Therefore, it is desirable to utilize real images to generate a super-resolved IRF-S for the proposed method.

## 5. Conclusions

In this study, our major objectives were successfully accomplished. The proposed SR scheme was effectively designed to have robustness at a high SR degree. In addition, proper preprocessing steps are contained in the proposed scheme to deal with various motions of targets. Experiments demonstrated the effectiveness of the proposed SR scheme using various metrics. The major conclusions can be summarized as follows:

(1) In terms of both restoration and improvement, the proposed scheme led to considerably improved spatial resolution of the target images for various types of targets, leading to clearer information on the principal scatterers.
(2) In particular, the proposed method exhibited excellent SR capabilities at a high degree of SR in terms of PSNR, SSIM, and CT compared with other SAR SR methods. This implies that the proposed method can extract highly precise and meaningful information regarding the targets represented in satellite SAR images.
(3) The concept of the proposed scheme can be easily extended to other satellite SAR systems such as ICEEYE, Capella, TerraSAR-X, and KOMPSAT-6 if the preprocessing steps are slightly adjusted depending on the characteristics of the SAR system.
(4) It is expected that the proposed scheme will also be useful for improving target recognition capability using satellite SAR images.

## Figures and Tables

**Table 1:** PSNRs for super-resolved image in Figure 6.

	d				
	2	2.5	3	3.5	4
Burg	30.39	29.08	28.17	27.72	27.65
MCM	**30.88**	29.01	28.23	27.58	27.44
BP	29.68	28.76	28.13	27.99	27.63
BPDN	29.71	28.76	28.13	27.99	27.63
Proposed	29.64	**29.61**	**28.27**	**28.62**	**28.04**

**Table 2:** SSIMs for super-resolved image in Figure 6.

	d				
	2	2.5	3	3.5	4
Burg	0.68	0.62	0.51	0.46	0.44
MCM	0.71	0.63	0.54	0.44	0.42
BP	0.72	0.63	0.62	0.56	0.56
BPDN	0.72	0.64	0.62	0.56	0.56
Proposed	**0.75**	**0.74**	**0.62**	**0.64**	**0.58**

**Table 3:** Standard deviations of BPDN algorithm.

	d				
	2	2.5	3	3.5	4
PSNR	$5.29\times 10^{-4}$	$2.88\times 10^{-5}$	$7.27\times 10^{-6}$	$1.19\times 10^{-5}$	$6.48\times 10^{-5}$
SSIM	$6.92\times 10^{-6}$	$4.28\times 10^{-6}$	$4.2\times 10^{-6}$	$3.27\times 10^{-6}$	$2.79\times 10^{-6}$

**Table 4:** PSNRs for super-resolved image in Figure 8.

	d				
	2	2.5	3	3.5	4
Burg	**29.74**	28.78	28.13	27.7	26.77
MCM	29.54	**29.06**	27.65	26.96	26.84
BP	27.69	28.12	26.14	26.45	26.82
BPDN	27.66	28.13	26.13	26.45	26.83
Proposed	28.93	28.17	**28.36**	**28.6**	**26.86**

**Table 5:** SSIMs for super-resolved image in Figure 8.

	d				
	2	2.5	3	3.5	4
Burg	0.75	0.63	0.62	0.58	0.47
MCM	0.75	0.67	0.64	0.47	0.46
BP	0.71	0.68	0.64	0.61	0.56
BPDN	0.71	0.68	0.64	0.61	0.56
Proposed	**0.75**	**0.74**	**0.74**	**0.72**	**0.69**

**Table 6:** PSNRs for super-resolved image in Figure 10.

	d				
	2	2.5	3	3.5	4
Burg	28.74	27.63	27.22	26.68	26.3
MCM	28.81	27.38	27.26	26.71	26.1
BP	28.76	27.77	27.21	26.96	26.31
BPDN	28.77	27.77	27.21	26.96	26.31
Proposed	**29.42**	**28**	**28.32**	**27.7**	**27.2**

**Table 7:** SSIMs for super-resolved image in Figure 10.

	d				
	2	2.5	3	3.5	4
Burg	0.77	0.74	0.71	0.67	0.67
MCM	0.79	0.74	0.7	0.68	0.67
BP	0.8	0.78	0.75	0.74	0.71
BPDN	**0.8**	0.78	0.75	0.74	0.71
Proposed	0.8	**0.79**	**0.76**	**0.77**	**0.77**

**Table 8:** CTs for super-resolved images in Figure 13.

	r				
	3	4	5	6	7
Burg (s)	0.15	0.22	0.29	0.39	0.48
MCM (s)	0.15	0.21	0.3	0.4	0.52
BP (s)	4.19	8.66	17.15	31.68	45.52
BPDN (s)	29.2	64.45	117.24	195.72	298.25
Proposed (s)	0.12	0.14	0.23	0.37	0.39

**Table 9:** SEs of super-resolved images in Figure 13 and Figure 14.

	Algorithm						
r	PR	REF	Burg	MCM	BP	BPDN	Proposed
4	9.74	8.82	7.64	7.94	7.63	7.62	7.13
7	9.74	8.82	7.03	7.75	6.51	6.52	6.69

**Table 10:** ICs of super-resolved images in Figure 13 and Figure 14.

	Algorithm						
r	PR	REF	Burg	MCM	BP	BPDN	Proposed
4	8.16	15.53	32.43	26.03	37.79	37.73	47.12
7	8.16	15.53	57.9	30.47	84.23	83.93	69.14

## Data Availability

Not applicable.
